# Case Report: Coexistence of an esophageal schwannoma disguised as a leiomyoma with a gastrointestinal stromal tumor of the gastric fundus

**DOI:** 10.3389/fonc.2025.1573436

**Published:** 2025-04-16

**Authors:** Yuedong Wang, Zhifei Xin, Wenbo Wu, Zhonghui Hu, Zhenghao Jia, Chengyao Zhang, Yi Ma, Xiaopeng Zhang

**Affiliations:** ^1^ Hebei Medical University, Shijiazhuang, Hebei, China; ^2^ Department of Thoracic Surgery Hebei General Hospital, Shijiazhuang, Hebei, China; ^3^ North China University of Science and Technology, Tangshan, Hebei, China

**Keywords:** esophageal schwannoma, leiomyoma, gastrointestinal stromal tumor, case report, differential diagnosis

## Abstract

To our knowledge, this is the first reported case of coexisting esophageal schwannoma and gastric fundus gastrointestinal stromal tumor (GIST). This case report describes the diagnostic and treatment process of a patient with esophageal schwannoma who also had a concurrent gastric fundus GIST and was presented to Hebei General Hospital (Hebei, China) in October 2024. The association between the pathogenesis of the two types of submucosal gastrointestinal tumors is unclear, with limited existing evidence in the literature. The esophageal schwannoma was misdiagnosed as a leiomyoma preoperatively, which prompted us to seek new diagnostic modalities to differentiate gastrointestinal submucosal lesions (leiomyomas, GISTs, and schwannomas). Surgical resection is considered the optimal treatment for esophageal schwannoma. The patient underwent a right single-port thoracoscopic esophageal tumor resection and recovered well, subsequently being discharged smoothly from the hospital.

## Introduction

The coexistence of esophageal schwannoma and GIST of the gastric fundus is a rare clinical scenario that poses significant diagnostic challenges. Schwannomas, typically benign nerve sheath tumors, and GISTs, which are mesenchymal tumors, are both submucosal gastrointestinal lesions. However, their coexistence has not been extensively documented in the medical literature. Patients diagnosed with esophageal schwannoma primarily present with symptoms such as dysphagia and difficulty breathing (1). The mechanism of the simultaneous occurrence of schwannomas and GISTs remains unclear at present, but relevant hypotheses have been proposed and require further exploration (2). It is difficult to differentiate schwannomas from GISTs and leiomyomas based solely on symptoms, signs, and imaging studies. Often, postoperative pathological examination and immunohistochemical staining are necessary to aid in diagnosis (3). Surgical treatment is the main therapeutic approach for this condition.

## Case report

**Figure 1:**
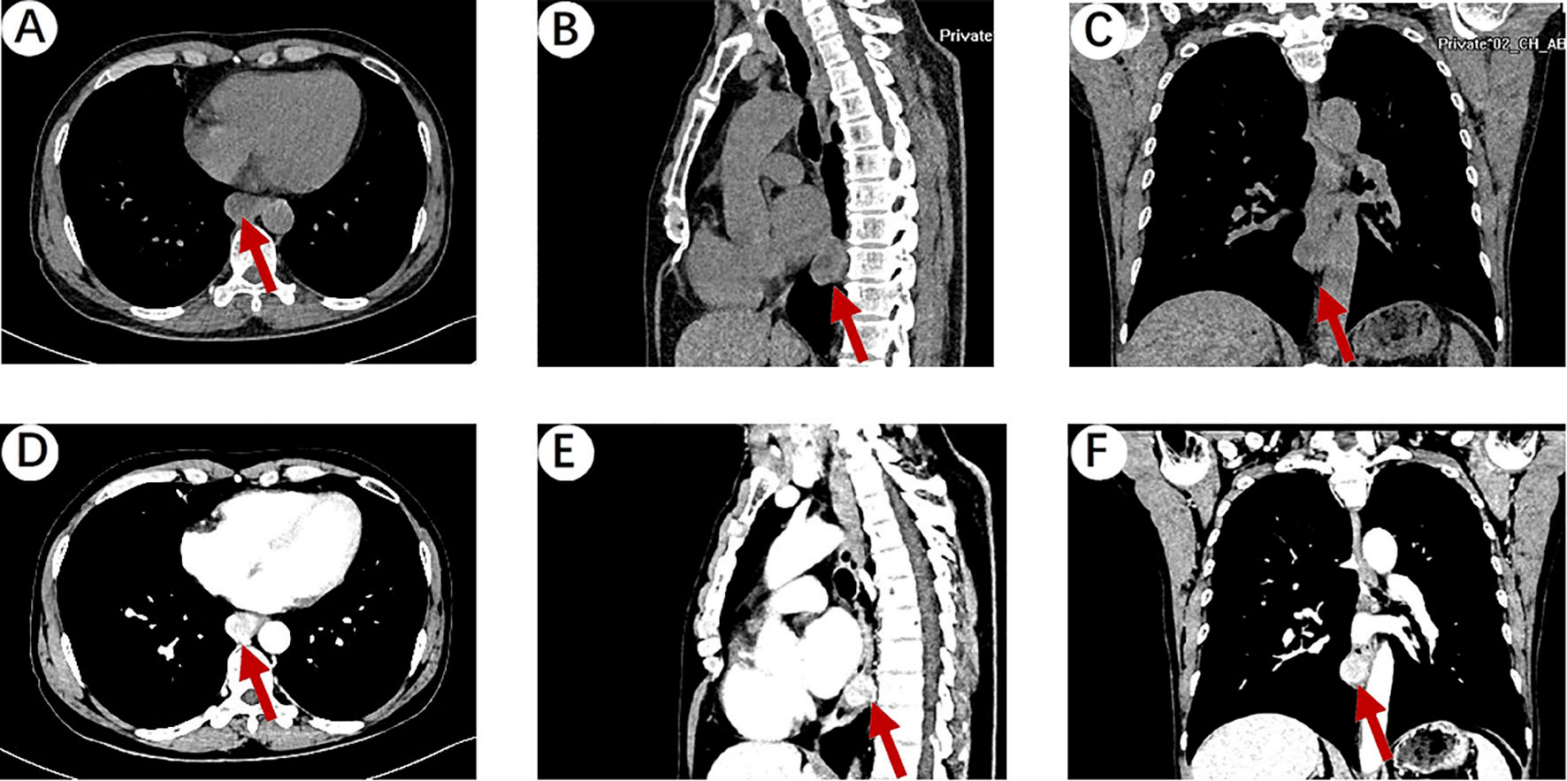
**(A)** Chest plain CT scan showing an ovoid soft tissue density shadow (red arrow), approximately 24×21×27mm in size, located at the level of the lower esophagus around the T9 vertebral body. The corresponding lumen at this level showed stenosis. **(B, C)** Sagittal and coronal reconstruction showing the ovoid soft tissue density shadow (red arrow). **(D)** The venous phase of the enhanced scan showed that the mass was marked but inhomogeneous enhancement, with a slightly higher enhancement degree than the adjacent esophageal wall. **(E, F)** Sagittal and coronal reconstruction of the venous phase of the enhanced scan showing the ovoid soft tissue density shadow (red arrow).

**Figure 2:**
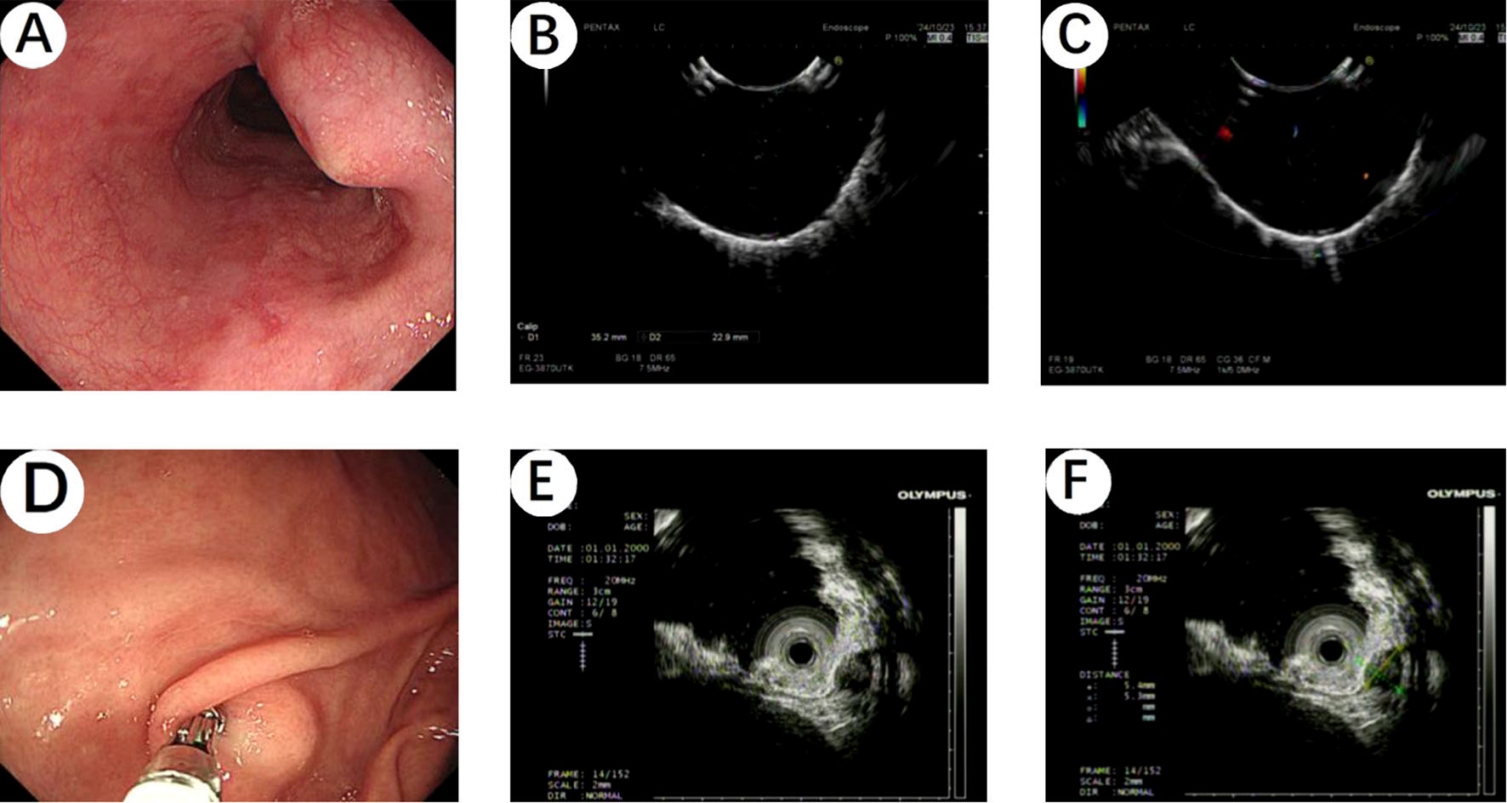
**(A)** Gastroscopy revealed a submucosal mass with a diameter of about 2.0cm at a distance of 30cm from the incisor, with a smooth surface. **(B, C)** Endoscopic ultrasound showed that the esophageal lesion was located in the muscularis propria, presenting as inhomogeneous hypoechoic, suggesting a leiomyoma. **(D)** A submucosal mass with a diameter of about 0.6cm was also visible on the greater curvature of the gastric fundus by gastroscopy, with a smooth surface. **(E, F)** The gastric fundus lesion was located in the muscularis propria, presenting as inhomogeneous hypoechoic, suggesting a stromal tumor.

A 64-year-old male patient presented to Hebei General Hospital on October 21,2024 with a chief complaint of persistent upper abdominal pain for 4 hours without a clear inciting factor. The patient’s upper abdominal pain is persistent, without any factors causing exacerbation or relief, no radiation pain, no nausea or vomiting, and no fever or diarrhea. A physical examination of the patient revealed tenderness in the upper abdomen. He had no significant medical history or family history of cancer. Upon admission, a computed tomography (CT) of the chest (**Figures 1A–C**) revealed an ovoid soft tissue density shadow, approximately 24×21×27mm in size, located at the level of the lower esophagus around the T9 vertebral body. The corresponding lumen at this level showed stenosis, with the enhancement scan (**Figures 1D–F**) showing marked but inhomogeneous enhancement. The CT values during the plain scan phase are 29 HU, and the CT values during the arterial phase and venous phase of the enhanced scan are 53 HU and 107 HU, respectively, with a slightly higher enhancement degree than the adjacent esophageal wall, and it was recommended to combine endoscopic examination. Further gastroscopy (**Figure 2A**) revealed a submucosal mass with a diameter of about 2.0cm at a distance of 30cm from the incisor, with a smooth surface. The mass felt hard when touched with biopsy forceps and was non-motile. A submucosal mass (**Figure 2D**) with a diameter of about 0.6cm was also visible on the greater curvature of the gastric fundus, with a smooth surface and hard texture when touched with biopsy forceps, and it was mobile. Endoscopic ultrasound (**Figures 2B, C**) showed that the esophageal lesion was located in the muscularis propria, presenting as inhomogeneous hypoechoic, with the largest cross-section measuring approximately 3.2×2.5cm, with clear borders, predominantly extraluminal growth, and located close to the aorta, suggesting a leiomyoma. The gastric fundus lesion (**Figures 2E, F**) was located in the muscularis propria, presenting as inhomogeneous hypoechoic, with the largest cross-section measuring approximately 0.5×0.6cm, with clear borders and intramural growth, suggesting a stromal tumor.

**Figure 3:**
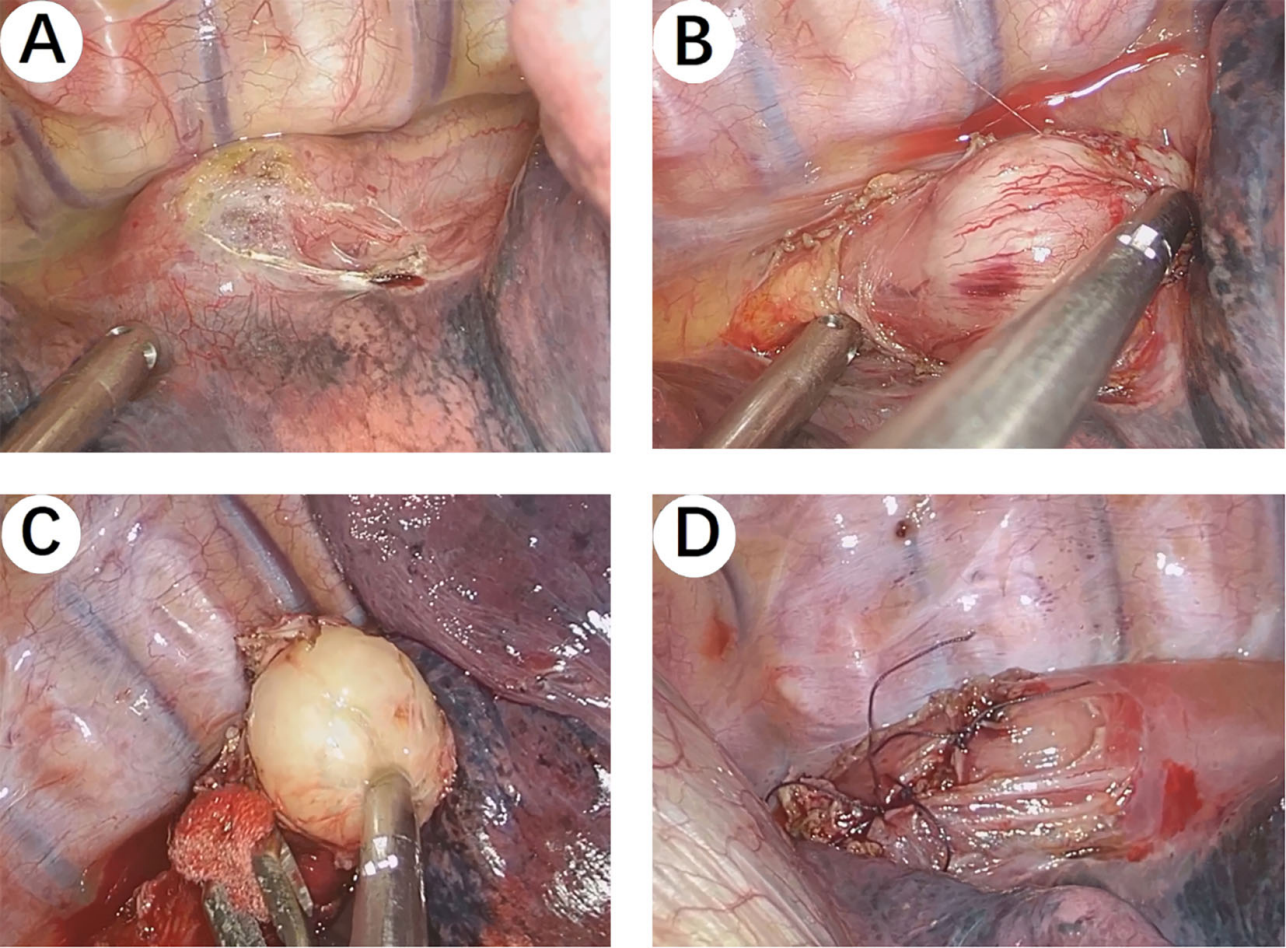
Intraoperative Images. **(A, B)** The esophageal lesion was found in the middle and lower part of the thorax, with a hard texture. No invasion of surrounding tissues was observed. **(C)** The tumor was carefully and completely excised. **(D)** The esophageal wound was sutured with silk sutures.

**Figure 4:**
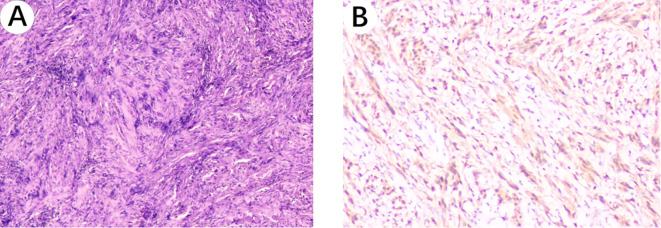
Histopathological images. **(A)** Microscopic image of the esophageal schwannoma taken through a 10x objective on the hematoxylin and eosin-stained histological section shows: a spindle cell tumor. **(B)** S100 positivity image of the esophageal schwannoma taken through a 100x objective.

**Figure 5:**
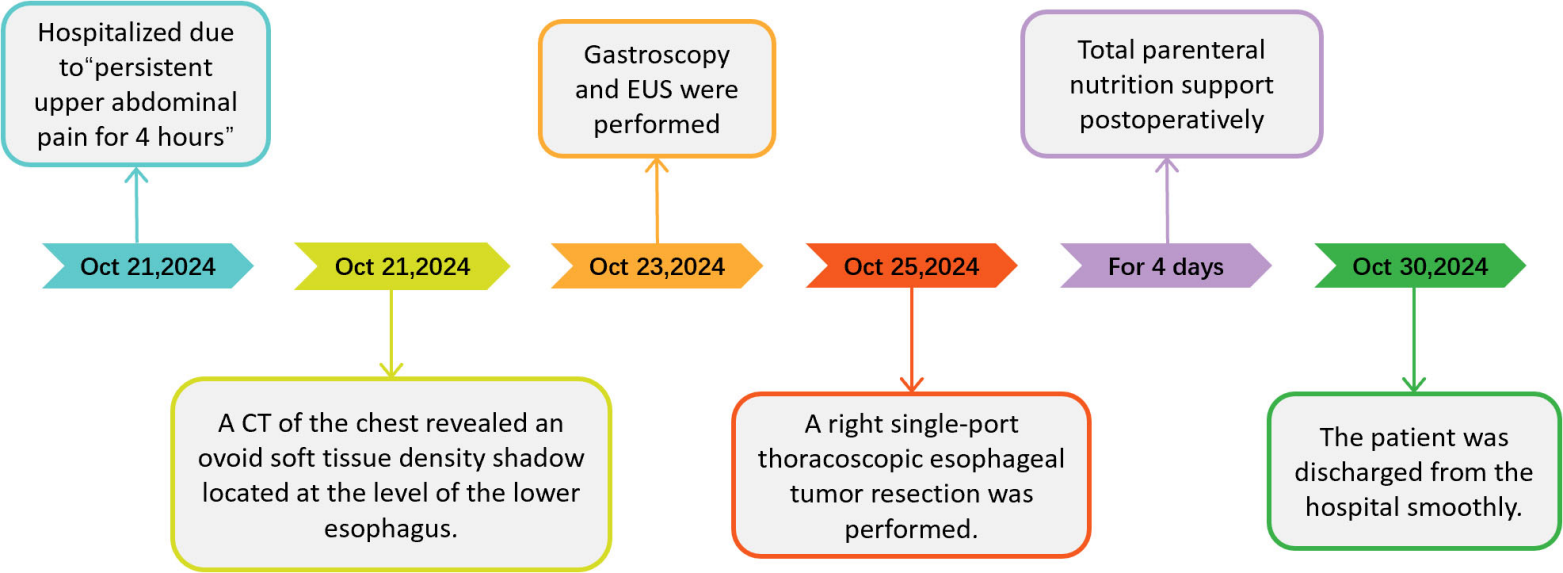
Diagnosis and treatment timeline of this case.

The patient underwent a right single-port thoracoscopic esophageal tumor resection on October 25, 2024. After successful general anesthesia, the patient was placed in a left lateral decubitus position. A thoracoscopic surgical incision of approximately 3cm was made at the 5th intercostal space along the posterior axillary line on the right chest. The skin, subcutaneous tissue, and muscle tissue were sequentially incised and bluntly separated to the pleural cavity. The esophageal lesion (**Figures 3A, B**) was found in the middle and lower part of the thorax, with a hard texture. No invasion of surrounding tissues was observed. Considering the preoperative gastroscopy results, a leiomyoma was suspected, and the tumor (**Figure 3C**) was carefully and completely excised. The esophageal wound (**Figure 3D**) was sutured with silk sutures, and the pleural cavity was irrigated with warm saline. The anesthesiologist was instructed to inflate the lungs, and no significant air leakage was observed near the esophagus. A fine drain was placed at the 7th intercostal space along the posterior axillary line, and the chest was closed in layers. The postoperative pathological report (**Figures 4A, B**) shows: a spindle cell tumor, which is consistent with the diagnosis of schwannoma based on immunohistochemical staining. Immunohistochemical staining: CD117 (-), CD34 (-), S100 (+), SMA (-), DOG-1 (-), Desmin (-), Ki-67 (5%+), Actin (-), SDHB (partly +), SOX-10 (+). This was inconsistent with the preoperative endoscopic ultrasound results and the judgment made during surgery. The patient received total parenteral nutrition support postoperatively and was gradually transitioned to oral feeding, making a good recovery. The chest X-ray was reviewed on October 28, 2024, and no significant abnormalities were found. He was successfully discharged on October 30, 2024. Three months after the surgery, we conducted a telephone follow-up with the patient, who reported no discomfort such as difficulty in swallowing. The patient has minimal concerns about the gastric fundus GIST and will undergo regular follow-up examinations as advised by the doctor. The diagnosis and treatment timeline for this patient is summarized in **Figure 5**.

## Discussion

A systematic review including 46 patients showed that esophageal schwannomas tend to occur in elderly individuals and females, with the most common site of occurrence being the thoracic esophagus (67.39%), and the most common symptom being dysphagia (69.56%). It is usually diagnosed postoperatively (76.08%) and is commonly treated with excision surgery (45.65%) (1). Benign esophageal tumors account for approximately two percent of all esophageal tumors. Over 80% of these tumors are leiomyomas (4, 5). Esophageal schwannomas are extremely rare. Chaterlin and Fissore described the first case of esophageal schwannoma in 1967 (6). The disease is more common in Asian population (3). Patients with esophageal leiomyomas may remain asymptomatic for a long time. Symptoms depend on the location and size of the tumor. The most common symptoms include progressive dysphagia (53.7%), dyspnea (10.4%), cough (4.4%), chest pain (4.4%), and weight loss (4.4%) (6). The typical differential diagnosis for schwannomas includes GISTs and leiomyomas (7). GISTs originate from the interstitial cells of Cajal, which are mesoderm-derived cells expressing the c-KIT protein (8). The most common symptoms are abdominal pain, gastrointestinal bleeding, and a palpable mass (9). For leiomyoma, at least half of the patients are asymptomatic and are often diagnosed incidentally during esophagogastroduodenoscopy. The most common symptoms are dysphagia, pain, and weight loss (10). Judging from these, we can reasonably deduce that the patient’s persistent upper abdominal pain is more likely to be caused by the GIST of the gastric fundus rather than the esophageal schwannoma.

Various hypotheses have been proposed to explain the coexistence of GISTs and schwannomas. Firstly, genetic mutations or combined gene dysregulation may contribute to the simultaneous development of both (11). Existing studies have shown that GISTs are associated with mutations in the c-KIT and PDGFRA genes, and the activation of their downstream pathways has been confirmed. c-KIT receptor activating mutations occur in 60-96% of GISTs. c-KIT exons involved in GISTs are 11, 9 and exons 13, 17, 18 (12). Although the mutation rate of PDGFRA is significantly lower than that of c-KIT (3.5% to 7.2%), it is also one of the mechanisms involved in the pathogenesis of GISTs (13, 14). The most common mutation in the PDGFRA gene is located in exon 18, where p.D842V accounts for 60 to 65%, and a few occur in exons 12 and 14 (2). Approximately 10-15% of GISTs do not have detectable mutations in any of these receptors (wild type), suggesting that other molecular pathways may also be involved in the development and progression of these tumors (15). Wild-type GISTs are categorized into succinate dehydrogenase (SDH) deficient and non-SDH deficient groups (16). The non-SDH deficient group includes neurofibromatosis type 1 (NF1) and GISTs with mutations in BRAF, KRAS, PIK3CA, and fusion genes (16). Previously, S. Charfi and Agaimy A have both separately reported the coexistence of GISTs and gastric schwannomas in patients with NF-1 (17, 18). NF-1 is a common genetic syndrome caused by mutations in the NF-1 gene located on chromosome 17 (17q11.2) (17). These pieces of evidence strongly suggest that the coexistence of schwannomas in the digestive tract with GISTs may be highly related to genetic mutations or combined gene dysregulation. Due to the rarity of cases, the types of genetic mutations in esophageal schwannomas have been rarely reported, which calls for further investigation in our subsequent work. Secondly, common initiating factors between different mesenchymal tumors may serve as the foundation for the pathogenesis of GISTs and schwannomas (7). Some carcinogens, such as N-methyl-N-nitro-N-nitrosoguanidine, are associated with the development of tumors that accompany GISTs in experiments (17). The preoperative diagnosis of esophageal schwannoma is difficult and often requires confirmation by immunohistochemical staining after surgery. On CT, there are no distinct features to differentiate schwannomas from other submucosal tumors (19). A fluorodeoxyglucose (FDG) -positron emission tomography (PET) scan is also not diagnostic, as both esophageal schwannomas and GISTs can accumulate FDG in submucosal esophageal lesions (20). Cold biopsy forceps were used to confirm the morphology and size of the tumor, as well as its mobility and consistency (21). Liquid biopsy is the latest non-invasive preoperative diagnostic technique with a promising future. Diagnosis is achieved through the analysis of circulating tumor DNA (ctDNA), circulating tumor cells (CTCs), free circulating nucleic acids, “tumor-educated platelets” (TEPs), and exosomes (22). Fine-needle aspiration biopsy (FNAB) under endoscopic ultrasound (EUS) guidance is very useful for preoperative diagnosis and management of the disease^4^. Some researchers believe that the current EUS-guided FNAB technique has a diagnostic accuracy of 52% to 86% in diagnosing submucosal esophageal tumors (23, 24). Of course, there have also been some encouraging advancements in diagnostic technology, and the progress of nanotechnology has greatly promoted the improvement of related diagnostic tools (12). In general, histological features of schwannoma include spindle-shaped tumor cells arranged in a palisading pattern or with loose cellularity in a reticular array (20). Esophageal schwannoma cells were positive for S100 protein but negative for smooth muscle markers such as actin and desmin, which were positive in smooth muscle tumors, while CD34 and CD117 were characteristically positive in GIST (25). The combined use of S100 and SOX-10 helped to improve the sensitivity and specificity of schwannoma diagnosis (3). They are usually benign and exhibit 1 or 2 histological patterns: Antoni A and B (26). Antoni A areas are compact zones with palisading of spindle cells, whereas loosely arranged tissue with variable cystic change and hemorrhage is designated Antoni B (27). There have also been reports of malignant schwannoma patients, but this condition is extremely rare (28).

Radiation therapy and chemotherapy are ineffective for esophageal schwannomas (29). Benign esophageal schwannomas typically require only tumor removal, not complete resection (3). Esophagectomy is performed when there is dense adhesion or when the tumor may involve the mucosa (6). Excision is the preferred method for smaller submucosal tumors, less than 2 cm in length, whether performed via thoracoscopy or endoscopically (5). Recently, it has become possible to remove giant esophageal schwannomas through robot-assisted excision (30). However, for larger tumors, usually greater than 8 cm in diameter and with extensive adhesions to the muscular layer, the resulting mucosal defect after resection can be quite large. In such cases, segmental esophagectomy followed by esophagogastrectomy is the preferred surgical approach (31). When malignancy is suspected, radical surgery is required, including esophagectomy and lymph node dissection, to avoid any recurrence or distant metastasis (27). Submucosal esophageal schwannoma excision via a thoracoscopic approach may sometimes lead to accidental injury to the esophageal mucosa, and to reduce the potential risk of mucosal injury during surgery, it may be chosen to perform an upper gastrointestinal endoscopy during the thoracoscopic surgery to ensure the integrity of the esophageal mucosa (3). Benign esophageal schwannomas generally have a good prognosis after complete resection, with a low recurrence rate (32). Surgery is the main treatment method for localized GISTs, which is typically performed using laparoscopic surgery (33). Patients with advanced GISTs treated with imatinib have significant survival benefits, with a 9-year survival rate ranging from 35% to 49% (34). At the same time, bio-nanomaterials offer promising opportunities for targeted drug delivery, overcoming treatment resistance, and improving therapeutic efficacy (12).

To our knowledge, this is the first case report of coexistence of an esophageal schwannoma and a GIST of the gastric fundus. The limitation of our study is that the submucosal mass in the greater curvature of the gastric fundus did not receive a definitive pathological diagnosis, but was only diagnosed clinically through EUS. Of course, we will continue to follow up the changes of the submucosal tumors at the gastric fundus of the patient.

## Conclusion

In summary, we describe a case of the coexistence of an esophageal schwannoma disguised as a leiomyoma with a GIST of the gastric fundus, and the successful treatment of the patient’s esophageal schwannoma via a right single-port thoracoscopic esophageal mass resection. This case report helps us to objectively recognize the clinical scenario of the coexistence of various submucosal gastrointestinal tumors, providing a reference for us to face the challenges in the diagnosis and treatment of such diseases and also motivates us to further investigate the pathogenesis of the coexistence of multiple submucosal gastrointestinal tumors.

## Data Availability

The raw data supporting the conclusions of this article will be made available by the authors, without undue reservation.
